# From Clinic to Classroom: Exploring the Journey of Healthcare Clinicians Entering Higher Education

**DOI:** 10.1111/tct.70557

**Published:** 2026-11-02

**Authors:** Paul Chesterton, Dawn Drury, Daniel Jones, Craig Tears

**Affiliations:** ^1^ School of Health and Life Sciences Teesside University Middlesbrough UK

**Keywords:** allied health education, clinical‐to‐academic transition, higher education workforce development, mentorship and support, pedagogical training

## Abstract

**Introduction:**

Transitioning from clinical practice to academia represents a significant career shift. Existing research has predominantly explored clinicians' transition experiences, with fewer studies examining evidence‐informed support strategies. This study aimed to generate evidence‐informed guidelines to facilitate effective transitions for newly appointed academic staff.

**Methods:**

A mixed‐method design, informed by role transition theory, comprised of a cross‐sectional survey of clinicians (*n* = 122) appointed to academic roles in the United Kingdom, followed by semi‐structured interviews (*n* = 14) to explore their transition experiences in greater depth.

**Results:**

Of 122 survey respondents, 38% (*n* = 46) reported prior teaching experience. While confidence in teaching within areas of clinical expertise was high, preparedness for broader academic responsibilities particularly assessment, curriculum development and institutional processes was variable. Integrated analysis from both the survey and qualitative data identified four interrelated themes: (1) disruption of professional identity and loss of established competence; (2) recalibration of expectations in response to the complexity and ‘invisible’ work of academia; (3) challenges in translating clinical expertise into pedagogical practice; and (4) the critical role of relational and structural support in facilitating identity development and belonging.

**Discussion:**

Quantitative findings were elaborated by qualitative data, revealing that early transition was characterised by uncertainty, imposter syndrome and experiential learning amid high workloads, diverse responsibilities and limited pedagogical preparation. Mentorship and peer support, where available, mitigated developmental gaps, highlighting the importance of structured induction, role clarity and pedagogical training to support staff readiness, retention and allied health education. Longitudinal research should evaluate the effectiveness of induction and mentorship programmes.

## 1 Introduction

Higher education institutions (HEIs) play a pivotal role in preparing health profession graduates who can effectively adapt, manage and innovate within a constantly evolving healthcare landscape. Pre‐registration healthcare programmes are responsible for equipping students with the essential clinical and non‐clinical skills, knowledge and professional behaviours needed for successful entry into a variety of healthcare professions [1]. A crucial component of preparing graduates is to immerse and expose them to contemporary practice, which can be achieved through firsthand experiences of clinicians who understand the challenges they will likely encounter. Students place significant value on the practical insights that clinicians contribute to both teaching and the overall educational experience [2]. This, combined with the requirement for teaching staff at HEIs to hold relevant healthcare qualifications and professional registrations, has created a career pathway for clinicians aspiring to educate the next generation of healthcare professionals.

In this study, the terms clinician/practitioner/healthcare professional refers to individuals registered as dietitians, midwives, nurses, occupational therapists, operating department practitioners, optometrists, paramedics, physiotherapists or radiographers. Key aspects of academic roles typically encompass teaching and learning approaches, assessment practices, marking and feedback, curriculum design, research and the navigation of university policies and regulations. Akkermans et al. [3] conceptualise a career shift as a ‘transition process during which an individual prepares for, undergoes, and adjusts to a significant change in work‐related content and context in their career’.

Internal pressures faced by HEIs, including academic staff shortages and a lack of doctoral‐level academics, have led to an increased reliance on staff from practice to support curriculum delivery, whether in a casual or full‐time role. While this approach benefits students by exposing them to current, frontline clinical expertise, casual and new academic staff often have limited familiarity with the broader curriculum structure, including course and module learning alignment and assessment literacy [4].

The transition from clinician to educator is frequently complex, with research identifying emotional, cognitive and professional challenges [5], and may be accompanied by anxiety, uncertainty and perceived inadequacy [6]. While this career shift may be motivated by a desire to teach, shape future professionals and pursue new professional opportunities [7], clinicians frequently enter academia with limited preparation for the pedagogical and institutional demands of the role. Access to collegial and institutional support during the early stages of transition is therefore critical in enabling individuals to navigate the demands of academic roles [6].

This transition can also be conceptualised as a process of professional identity reconstruction, rather than a simple change in role or setting. Clinicians entering academia often possess well‐established professional identities grounded in expertise, autonomy and credibility within practice environments, though these forms of legitimacy do not always translate directly to academic contexts [8]. Ashforth's [9] transition theory suggests that moving into a new role involves a process of leaving a previous identity and developing a new one. During this transition, individuals may experience uncertainty (liminality), but as they gain confidence and authenticity in the new role, they become more committed to it. Successful role adaptation is supported by a sense of identity, meaning, belonging and autonomy. Individuals must adapt to new expectations, including pedagogical practice, scholarly activity and institutional responsibilities [10]. This transition is frequently characterised by uncertainty, reduced confidence and a perceived loss of competence, as individuals move from positions of expertise to relative novice status within an unfamiliar professional culture [5, 6]. This study adopted a professional role transition lens, informed by Ashforth's Role Transition in Organisational Life theory [9], to conceptualise and interpret the shift from practice to academia, exploring individual preparedness to broader processes of identity development and socialisation within higher education.

Despite the growing recognition of these challenges, existing literature remains largely descriptive, with a predominant focus on early‐stage transition experiences [11]. Consequently, there is limited understanding of how transitions can be systematically supported or optimised within institutional contexts [12], constraining the development of evidence‐informed strategies to support new academic staff and sustain educational quality. In addressing this gap, this study moves beyond descriptive accounts to generate applied, evidence‐informed guidance to support clinicians entering and establishing themselves within academic roles. This study was guided by two research questions: (1) What key challenges do clinicians experience when transitioning into academic roles, particularly in relation to professional identity, role expectations and institutional context? and (2) What evidence‐informed strategies can support sustained development and transition into higher education?

## 2 Methods

### 2.1 Design

A mixed‐methods study was conducted using a sequential explanatory design, comprising a cross‐sectional online questionnaire followed by semi‐structured interviews [13, 14]. Eligible participants were United Kingdom (UK) academics in their first teaching role for less than 18 months at survey release. As part of the survey, respondents could choose to provide consent to be contacted for a follow‐up semi‐structured interview. Data collection was conducted between October and January 2025. Ethical approval was granted by the School of Health and Life Sciences Ethics Committee at Teesside University (ID23297). Reporting follows STROBE guidelines [15].

### 2.2 Measures

The mixed‐methods approach facilitated respondents' sharing of experiences with deeper insights illuminated from qualitative components, including semi‐structured interviews [14]. Stage 1: An online cross‐sectional survey included quantitative questions with dichotomous, multiple‐choice and fully labelled unipolar Likert scales (1 = *strongly disagree* to 5 = *strongly agree*). A qualitative component featured open‐ended questions with text boxes to capture detailed, unrestricted responses.

A pilot group of nine UK academics with healthcare backgrounds reviewed the survey for content validity. They provided feedback on format, content, wording, functionality. This review process led to three rounds of revisions where six questions were removed, seven added, wording simplified and items reordered. Pilot participants were excluded from the final survey.

The final online survey, hosted on Onlinesurveys.ac.uk, included 41 core questions across 14 pages (see Supplementary Material 1). Branching logic directed participants based on prior responses, so not all questions applied to every respondent. The survey comprised four sections, (1) demographics and professional experience, (2) perceptions of and preparedness for an HE role, including enablers and barriers, (3) learning and teaching practices which covered assessment, marking and feedback, curriculum development and personal tutoring, and (4) future career aspirations.

Stage 2: Participants could self‐select for a follow‐up online semi‐structured interview. To maintain anonymity, interested respondents submitted emails via a separate link, removing any connection between their identity and survey responses.

#### 2.2.1 Procedure

Study recruitment messages and emails were disseminated to several professional healthcare groups, (e.g., British Dietetic Association HE group, Chartered Society of Physiotherapy's interactive community, Royal College of Occupational Therapists, Royal College of Paramedics, UK Heads of Radiography Forum and Undergraduate Optometry Course Leaders Forum) and UK Higher Education Networks, with additional snowball sampling encouraging respondents to share the survey [16].

Respondents received details of the study's aims, estimated completion time and the storage of anonymised data. They were asked to complete the questionnaire once and to refrain from submitting multiple entries; however, this was not monitored or controlled on the online platform (e.g., through cookies, IP checks or registration requirements). Respondents could review and modify their answers by using the survey platform's ‘back’ option. By completing and submitting the survey, participants were informed that they were providing consent to participate. No incentives were offered for completing the survey.

Interviews, lasting approximately 30 min, were conducted online at participants' convenience by a designated researcher and recorded for transcription. Interviews were transcribed verbatim by a member of the research team (P.C.) and cross‐checked against the original audio recordings to ensure accuracy and completeness prior to analysis Interviews were guided by a semi‐structured framework approved by the pilot group (Supplementary Material 1), allowing both comprehensive coverage of key topics and flexibility to explore emergent issues [17].

#### 2.2.2 Data Analysis

Following survey closure, data were imported into Microsoft Excel (Microsoft, 2016). Dichotomous and multiple‐choice responses were converted to proportions with 95% confidence intervals using the Wilson procedure [18], while Likert responses were treated as numeric. Data were visualised in R‐Studio using flat‐violin and raincloud plots with overlaid boxplots to show medians and interquartile ranges, providing a comprehensive representation of the data distribution [19].

Quantitative and qualitative findings were analysed separately and subsequently integrated during interpretation using a sequential explanatory approach [13, 14]. Areas of convergence, complementarity and expansion were examined to develop an overarching understanding of clinicians' transition experiences. Qualitative findings were used to explain and contextualise patterns identified in the survey data.

A thematic analysis of qualitative survey and interview data was conducted using a six‐phase process to identify patterns in participants' perceptions, experiences and knowledge [20]. The analysis was not a linear process of moving through the phases, but rather a recursive and inductive process, of moving back and forth as necessary [21]. Coding was collaborative and reflexive, focusing on generating nuanced understanding and patterns of meaning through data familiarisation, coding and iterative theme development [22]. Data were exported to Microsoft Word (Microsoft, 2016) for familiarisation by two researchers (P.C. and C.T.).

Cognisant of positionality, the two researchers have lived experience in transitioning from Physiotherapy practice to full time academic roles. Reflexivity accounts for the researchers subjectivity and is integral to the analysis process. Maintaining a reflexive journal and peer discussion was engaged to enhance transparency and methodological rigour [23].

Comments were manually coded (labelled) into initial categories, which were reviewed and refined over two coding sweeps to generate main and sub‐themes through an iterative mapping process. Themes were organised around central organising concepts [22]. After independent analysis, the two researchers held a debriefing session to discuss and refine findings. No disagreements arose during coding; however, a third author was available if needed. Theoretical saturation was achieved when data were sufficient to replicate the study, with no new insights emerging, enhancing trustworthiness. Triangulation of codes confirmed convergence between researchers' conclusions, strengthening credibility, validity and trustworthiness [23]. Themes and relationships to data were reviewed by author D.D.

## 3 Results

### 3.1 Respondent Characteristics

A total of 122 survey responses were analysed following exclusion of three ineligible participants. Fourteen interviews were completed from 27 volunteers. Respondents represented allied health professions (Supplementary Materials 2), with a mean of 14.5 years clinical experience (SD 7.9). Most held full‐time academic posts (61%), with 52% maintaining clinical practice. Over half held Master's qualifications (52%), and 38% had completed a teaching qualification prior to appointment. The median interval to first teaching was under 5 weeks.

### 3.2 Overview of Findings

Analysis identified four themes that address the study's two research questions, capturing both the challenges associated with transition and the mechanisms through which clinicians adapt to academic roles. These themes reflect interrelated processes of role exit/entry and identity disruption, expectation recalibration, pedagogical development and the role of institutional support.

### 3.3 Theme 1: Disruption of Professional Identity and Loss of Established Competence

Transition from clinical practice to academia disrupted participants' professional identity by challenging the legitimacy of expertise that had previously underpinned their confidence, autonomy and professional status. Rather than representing a simple role change, transition required clinicians to renegotiate what it meant to be competent within an unfamiliar academic context. Despite extensive clinical experience, clinical expertise alone was insufficient to establish academic credibility, resulting in uncertainty and diminished confidence.

*Transition from clinical practice to academia disrupted participants' professional identity*.

At first, I had no idea what I was doing … through trial and error, I got there. (Participant 09)

The disruption stemmed from a misalignment between clinical and academic forms of legitimacy. Credibility within academia was associated with pedagogical knowledge, assessment literacy and institutional fluency, rather than clinical expertise. Consequently, participants experienced a disconnect between what they knew and what was valued, contributing to uncertainty and reduced confidence. Imposter syndrome was commonly reported and appeared to be reinforced by the absence of clear expectations and limited formative feedback.

You doubt yourself because there's no feedback until students complain. (Participant 13)

Unlike clinical environments, where feedback was immediate and embedded within practice, academic feedback was often delayed and evaluative. This shift limited opportunities to calibrate performance during early transition, reinforcing uncertainty and perceptions of diminished competence. Participants recounted a temporary regression from expert clinician to novice academic, requiring renegotiation of competence and professional belonging. Improvement occurred primarily through self‐directed learning and experiential engagement rather than structured institutional support.

Findings conceptualise transition as an identity reconstruction process, in which clinicians renegotiate professional legitimacy within a new epistemic and organisational context. The absence of structured support during this phase may prolong uncertainty and delay the development of a secure academic identity.

### 3.4 Theme 2: Navigating Invisible Work and Recalibrating Expectations of Academic Practice

Transition required clinicians to recalibrate their understanding of academic work as they encountered responsibilities that extended well beyond pre‐entry expectations. Rather than workload alone, it was the hidden complexity of academic practice that challenged assumptions about what the role involved and what constituted academic work.

I thought I would just be teaching all day. There is so much more to it! (Participant 06)

Academic roles were constructed as multi‐dimensional, encompassing administrative, pastoral and organisational responsibilities that were often unanticipated. These aspects were conceptualised as ‘invisible work’, reflecting their absence from pre‐entry expectations despite being central to academic practice.

It feels like I'm doing three jobs: admin, support, and teaching. (Participant 09)

The resulting mismatch exposed an epistemic gap between individuals' assumptions about academic work and the realities of institutional practice. Participants were required to navigate complex organisational systems, often experienced as opaque and difficult to access.

Navigating the systems here is like learning a new language. (Participant 07)

Workload was reported as both intensive and unpredictable, with cumulative demands extending beyond allocated workload models. Participants' accounts suggested that the challenge lay not only in workload volume but also in the cognitive fragmentation created by competing teaching, administrative and pastoral responsibilities.

There's just not enough hours in the day … ..it takes over your evenings and weekends. (Participant 04)

These demands contributed to stress, reduced control and emotional strain, often extending into personal time.

You dread having holidays because it's literally like, what will I come back to. (Participant 01)

Recalibrating expectations therefore involved more than recognising additional responsibilities; it required participants to reconstruct their understanding of academic work and its professional demands. While some developed greater appreciation for the broader purpose of academic roles, others experienced disillusionment and questioned long‐term sustainability.

If I'm honest, I don't feel the job satisfaction that I thought I would. (Participant 02)

Despite these challenges, most respondents (73%) intended to remain in academia, although only 61% reported alignment between expectations and experience. This apparent contradiction suggests that commitment to teaching frequently persisted despite disappointment with organisational realities, reflecting an ongoing negotiation between intrinsic motivation and institutional demands.

These findings position transition as a process of expectation realignment in which clinicians reconcile professional motivations with the structural realities of academic work. Where this reconciliation remained incomplete, tensions between role demands and professional values appeared to undermine satisfaction and potentially longer‐term retention.

### 3.5 Theme 3: Translating Clinical Expertise Into Pedagogical Practice

Transition required clinicians to translate, rather than simply transfer, their professional expertise into pedagogical practice. Although most participants taught within their area of clinical specialism (77%), disciplinary knowledge alone did not confer confidence or competence as educators.

Teaching is such a different skill … something I didn't really appreciate. (Participant 04)

Participants' accounts suggested that expertise was context dependent: confidence developed through clinical practice did not readily transfer to teaching, which demanded distinct pedagogical knowledge, assessment literacy and educational judgement. While participants reported confidence in subject knowledge, this did not equate to confidence in teaching, particularly during early transition. Those teaching outside their discipline (36%) reported lower competence.

Confidence in teaching remained fragile during early transition, shaped by limited preparation and the absence of structured guidance.

There was very little training, and I was just expected to get on with it. (Participant 06)

Learning was largely informal and experiential, relying on observation, peer interaction, and trial and error.

Over time, through watching others and trial and error, I got there. (Participant 09)

Survey data supported this pattern, with most participants independently developing learning materials (52%) and only minimal supervision reported. Confidence increased over time, indicating that pedagogical competence emerged primarily through experience rather than structured educational development.

Assessment literacy illustrated the limits of transferring clinical expertise into academic practice. Approximately half of participants reported no formal training, with only moderate confidence in aligning assessment with learning outcomes. Learning was described as tacit and socially mediated, often occurring through informal collaboration.

I was asked to mark straight away but had no idea how to do it. A colleague double‐marked with me, which helped. (Participant 13)

Similarly, most participants undertook personal tutoring roles (84%), demonstrating that while interpersonal skills transferred from clinical practice, institutional and pedagogical knowledge did not. Engagement in curriculum design (71%) reflected a similar pattern of partial preparedness, requiring participants to acquire new forms of educational expertise.

Overall, transition involved reconstructing professional expertise within an educational context by integrating clinical and pedagogical identities. Rather than representing a straightforward transfer of existing competence, becoming an educator required the development of new pedagogical capabilities that were acquired iteratively but received limited formal institutional support.

*Becoming an educator required the development of new pedagogical capabilities*.

### 3.6 Theme 4: Relational and Structural Support as Catalysts for Identity Reconstruction and Belonging

Transition was shaped by the extent to which clinicians were able to access relational and structural support that enabled participation in academic communities of practice. Rather than functioning solely as a supplementary resource, support operated as a mechanism through which identity reconstruction and belonging were made possible. Institutional support emerged as a critical factor in shaping transition, though it was inconsistently experienced. While most participants reported access to mentoring (84%), the quality and accessibility of support varied substantially.

If I'd had someone to ask the ‘stupid questions’, it would've eased the stress a lot. (Participant 11)

Support structures, including mentorship, induction and peer relationships, were experienced as key mediators of early transition, influencing both competence development and emerging academic identity. Where accessible, these facilitated clearer expectations, increased confidence and a greater sense of belonging. However, support provision was often misaligned with need, particularly during early transition. Induction processes were frequently delayed or generic, limiting their effectiveness.

*Support structures, including mentorship, induction and peer relationships, were experienced as key mediators*.

We need an induction that happens when the person starts – not 6 months later. (Participant 08)

As a result, support was often experienced reactively rather than proactively, requiring participants to navigate challenges independently before receiving guidance. Relational support, particularly from peers, was highly valued but not consistently available.

Having a mentor was helpful, but they were too busy to really support me. (Participant 14)

Informal networks were often more effective than formal mechanisms, highlighting gaps in institutional provision. The absence of opportunities for experiential learning, such as shadowing and feedback, further contributed to uncertainty.

I would have benefited from seeing someone else teach first—not just being thrown in to deliver a session. (Participant 04)

These accounts indicate that support functioned less as a discrete intervention and more as an infrastructural condition for learning within academic practice. In this sense, access to legitimate participation in academic communities of practice was unevenly distributed. Where such support was limited, participants described isolation and reduced confidence, reinforcing that transition is socially and institutionally co‐constructed. Effective transition is therefore contingent on the availability of timely, accessible and contextually aligned support structures that facilitate both learning and belonging.

## 4 Discussion

The findings extend existing literature by demonstrating that challenges in preparedness, workload and pedagogical development reflect levels of liminality through disruption to professional identity and legitimacy as clinicians transition into academic roles. Viewing transition through a role transition lens provides a coherent explanation for why experienced clinicians reported uncertainty, reduced confidence and imposter syndrome despite extensive clinical expertise. Transition therefore involves movement from recognised clinical expertise to novice academic status, requiring individuals to renegotiate competence, credibility and belonging [10].

While respondents were motivated by applying previous expertise and pursuing professional development, most entered teaching with limited academic experience and minimal pedagogical preparation. Despite the availability of teaching qualifications, early development relied largely on informal, experiential learning. These findings extend prior work by demonstrating that challenges in preparedness and early development reflect a misalignment between established professional expertise and emerging academic expectations of competence.

### 4.1 Preparedness as an Expression of Identity Disruption

Many academics described entering their roles with limited preparation and misconceptions about academic work, an experience widely echoed across nursing, midwifery and allied health literature [8]. Rather than reflecting a simple training deficit, preparedness reflected the need to reconstruct competence according to academic rather than clinical practice norms.

Although motivations such as applying clinical expertise, pursuing new challenges and improving work–life balance are well‐documented drivers of transition, our data suggests these expectations often collide with the realities of academic autonomy, competing demands and structural complexity. This misalignment helps explain why transition to the academic role is experienced as identity disruption rather than simply inadequate preparation. While existing literature highlights the importance of transparent role expectations, structured induction and sustained mentorship [24], the present findings suggest such support remains insufficiently aligned with the realities of transition. Conceptually, this reflects movement across fundamentally different professional cultures, requiring clinicians to renegotiate competence and credibility within new institutional logics [8, 25]. While previous work has acknowledged this shift [10, 26], the present study extends this, showing how clinical expertise is experienced as insufficient within academic settings, disrupting previously secure identities.

While alignment between clinical expertise and teaching responsibilities enhanced participants' confidence, this knowledge did not readily translate into pedagogical competence [25, 27]. Participants relied heavily on experiential and informal learning, highlighting an uneven and largely unsupported development process. Inconsistent structured support, alongside reliance on peer learning, mirrors existing evidence [10], but our findings demonstrate how such learning is shaped by identity negotiation rather than formal development pathways alone. Taken together, these findings suggest that pedagogical development is not simply the acquisition of teaching skills but a mechanism through which clinicians reconstruct their professional identity as educators.

Although participants engaged in curriculum development and evaluation, many felt underprepared, reflecting the specialised nature of this work. This aligns with broader evidence on the need for structured capacity‐building [28], but further suggests that curriculum‐related challenges reflect a deeper misalignment between disciplinary expertise and educational practice.

### 4.2 Clinician‐to‐Academic Transition as Professional Identity Reconstruction

The preceding findings suggest that clinician‐to‐academic transition is most usefully understood as a process of professional identity reconstruction. The transition from practice to academia represents a fundamental career shift rather than a simple role change, requiring reconstruction of professional identity while acquiring new academic skills and navigating unfamiliar institutional norms. Recognising this as a career transformation is essential for developing targeted strategies that support successful adaptation and retention. As Smith and Boyd [8] note, many clinicians retain a strong identification with their clinical roles, which can hinder the confident adoption of academic identities as scholars and researchers. Those who continue to practise clinically alongside their academic role may retain a sense of professional credibility that functions as a transitional bridge between the two identities [9]. Further investigation in this area could identify whether this hinders or enables the integration of a new role identity and long‐term sustainability of a career in academia.

*The transition from practice to academia represents a fundamental career shift*.

This identity‐based role transition interpretation provides a coherent explanation for the mismatch between expectations and the realities of academic work. The present findings show how structural tensions are experienced as cultural dislocation and identity uncertainty. Despite many reporting access to mentoring or induction, support was often superficial; new academics described navigating complex institutional systems largely alone. Effective mentorship demands structured, sustained and personalised engagement [29], yet such conditions were inconsistently realised.

### 4.3 Induction and Support as Mechanisms for Identity Development and Belonging

Timely and accessible induction is critical for a successful transition into academia [30]. Our data indicates that current practices, often delayed and inconsistently structured, left new staff underprepared at a pivotal juncture. Participants strongly advocated for an induction process that is immediate, structured and responsive to individual entry points, rather than determined by cohort‐based scheduling. From a role transition perspective, induction and mentorship function as mechanisms through which legitimacy, competence and belonging are negotiated, rather than simply as vehicles for knowledge transfer. Support therefore functions not only as a developmental resource but also as a mechanism through which newcomers gain legitimate participation within academic communities and begin to see themselves as academics. While participants emphasised the value of structured onboarding, clear expectations and access to institutional systems, the findings suggest that these elements are most effective when embedded within ongoing, relational support structures.

Beyond induction, participants described collegial support, practical exposure and ongoing professional development as essential for navigating the complexities of academic life. Structured support and a deliberate acculturation process enable new academics to develop teaching identities through formal and informal means [24]. While increased freedom can be empowering, it may undermine cohesion and teamwork for some. A responsive transition framework, combining immediate access to institutional knowledge with a supportive culture, is therefore critical for fostering early success and long‐term retention.

These findings suggest that effective transition requires more than front‐loaded induction. A valuable induction process necessitates sustained, context‐sensitive and relational support mechanisms that foster participation within academic communities of practice. Such an approach shifts induction from a discrete intervention to an ongoing process of identity formation and belonging, with important implications for staff development and retention.

### 4.4 Implications for Practice: Supporting Identity‐Conscious Transition

If clinician‐to‐academic transition is understood as a process of professional identity reconstruction, support strategies must extend beyond skills training to facilitate competence, legitimacy and belonging. Drawing on insights from this study, we propose targeted recommendations to strengthen the transition and ongoing development of clinical professionals into academia (Figure 1). For HEI's, this includes timely, structured induction, clear role expectations and targeted pedagogical development. Line managers play a critical role in providing sustained mentorship and facilitating integration into academic practice.

**FIGURE 1:**
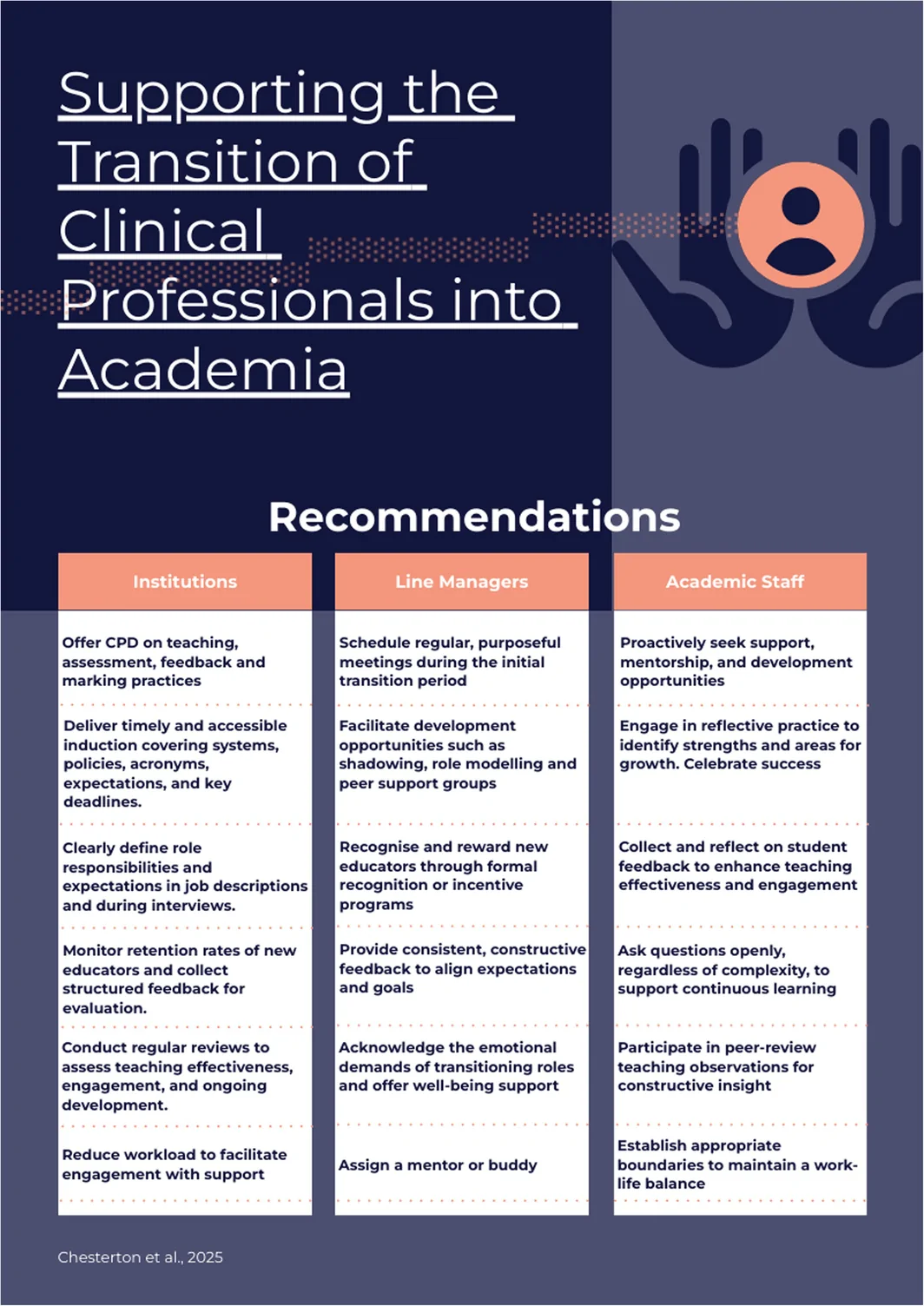
Recommendations for supporting the transition of clinical professionals into academia.

Clinical academics themselves are encouraged to engage in reflective practice and actively develop pedagogical and curriculum‐related competencies. Collectively, successful transition requires coordinated institutional and individual approaches that support ongoing identity development, confidence and retention.

### 4.5 Limitations

The sample may not fully represent the wider staff population, introducing the potential for responder/non‐responder bias [31]. While the survey instrument was piloted, formal measures of reliability were not assessed. While interviews provided valuable depth and context, participants may have self‐selected based on strong views or experiences, which may not reflect broader perspectives. As the study was conducted within the UK higher education context, findings may not be transferable to other settings. The absence of detailed demographic data restricts consideration of factors such as nationality or prior educational experience, which may influence transition experiences.

## 5 Conclusion

The transition of allied healthcare professionals into academia is a critical workforce challenge. Many enter academic roles underprepared, facing steep learning curves that threaten retention and the quality of health professions education. This study suggests that the diverse challenges reported during clinician‐to‐academic transition including preparedness, pedagogical development, workload and support are best understood as interconnected elements of professional identity reconstruction. This conceptualisation moves beyond describing transition challenges to providing a unifying explanation for how clinicians adapt to academic practice. Supporting these transitions is essential not only for individual success but also for building a sustainable, skilled academic workforce capable of meeting the growing demands of healthcare education.

## Author Contributions

**Paul Chesterton:** conceptualization, investigation, writing – original draft, methodology, writing – review and editing, validation, formal analysis, project administration, data curation. **Dawn Drury:** methodology, data curation, writing – review and editing, validation. **Daniel Jones:** methodology, data curation, formal analysis, writing – review and editing. **Craig Tears:** data curation, formal analysis, methodology, writing – review and editing.

## Funding

The authors have nothing to report.

## Ethics Statement

All procedures performed in studies involving human participants were in accordance with the ethical standards of the institutional and/or national research committee and with the 1964 Helsinki Declaration and its later amendments or comparable ethical standards. Ethical approval was granted from the School of Health and Life Sciences ethics committee at Teesside University (ID23297).

## Consent

Informed consent was obtained from all individual participants included in the study.

## Conflicts of Interest

The authors declare no conflicts of interest.

## Supporting information


**Data S1:** Supporting information.

**Data S2:** Supporting information.

## Data Availability

The data that support the findings of this study are not openly available due to reasons of sensitivity and are available from the corresponding author upon reasonable request.
