# Supplementary material for: From Clinic to Classroom: Exploring the Journey of Healthcare Clinicians Entering Higher Education
Source: Clin Teach. 2026 Nov 2;23(6):e70557. doi: 10.1111/tct.70557 (PMC13727958; doi:10.1111/tct.70557)
Supplement: Supplementary file 1 [file TCT-23-e70557-s001.docx]

**Supplementary Materials 2 – Respondents Clinical Profession**

| **Profession** | **N (%, 95 CI)** |
| --- | --- |
| Dietetics | 14 (11, 7 to 18) |
| Midwifery | 12 (10, 6 to 16) |
| Nursing | 23 (19, 13 to 27) |
| Occupational Therapy | 11 (9, 5 to 15) |
| Operating Department Practitioner | 9 (7, 4 to 13) |
| Optometrist | 8 (7, 3 to 12) |
| Paramedic | 13 (11, 6 to 17) |
| Physiotherapy | 16 (13, 8 to 20) |
| Radiographer | 16 (13, 8 to 20) |
