# Supplementary material for: From Clinic to Classroom: Exploring the Journey of Healthcare Clinicians Entering Higher Education
Source: Clin Teach. 2026 Nov 2;23(6):e70557. doi: 10.1111/tct.70557 (PMC13727958; doi:10.1111/tct.70557)
Supplement: Supplementary file 2 [file TCT-23-e70557-s002.docx]

**Survey Questions**

**Section 1:**

**1. Participant Demographics /Level of Experience:**

- Is your academic role full time or part time?
- Do you still work in a clinical role?
  - Is this in your own time or as a lecturer/practitioner post
- Which healthcare profession are you qualified in? (e.g., physiotherapy, occupational therapy)

**1.1 Discipline Experience:**

- How many years have you worked in your specific health related discipline or field? (i.e. pre-academic position)

**1.2 Qualifications:**

- What is your highest academic qualifications (e.g., BSc, MSc, PhD)?
  1.3 **Teaching Qualifications:**
- Have you had experience of teaching in Higher Education prior to taking your current role?
  - What academic level of students (undergraduate, postgraduate) did you teach in your previous role?
  - What academic level of students (undergraduate, postgraduate) did you teach in your previous role?
- Have you completed or are you working towards completing a higher education teaching qualification or equivalent?
- If you have 'completed' a higher education teaching qualification, how much do you agree with the following statement:
  - 'My teaching qualification has been effective for my academic teaching practice'?
- If you have 'completed' a higher education teaching qualification, how much do you agree with the following statement:
  - 'My teaching qualification has been effective for my academic teaching practice'?

**1.5 Current Teaching Experience:**

- Did you receive an induction when you started your current role?
- How well did your induction prepare you for your current role?
- Approximately how long had you been in this post for before you were asked to teach for the first time?
- Did you receive any teaching training before educating university students for the first time?
- Did you receive any support or mentorship from the faculty?

**Section 2:**

**2. Perceptions/Preparedness**

- What were your motivations for pursuing a career in academia?
- How prepared did you feel about teaching your first class?

**2.1 Enablers:**

- What has supported your transition into HE? What other support would you have wanted to receive?
- What percentage of your teaching is directly aligned to your area of expertise or specialty?
- If you teach outside of your direct speciality, how competent do you feel to teach that subject?
- How much do you agree with the following statement:
  - 'Drawing on my clinical experience has increased and impacted upon my teaching effectiveness?

**2.2 Barriers:**

- What challenges have you faced in your transition into HE? Please explain why
- How experienced are you with teaching technologies (e.g., simulation software, virtual labs, online classrooms)?
- How easy was it for you to adapt to the rules and policies of your institution regarding teaching?

**Section 3:**

**3.1 Learning and Teaching Approaches:**

- How do you plan and organise learning strategies within the modules you teach?
- How confident are you at planning and organising learning approaches within the modules you teach?
- Have you explored different teaching methods or approaches to enhance student learning? If so which ones?
- Do you create supplementary learning materials (e.g., online resources, handouts) for your students?
- How confident are you/were you at creating supplementary learning materials within the modules you teach?
  - On starting your post
  - Currently

**3.2 Assessment Planning:**

- Have you received any training on how to plan assessments?
- How confident do you feel about planning assessments that align with learning outcomes?

**3.3 Marking and Feedback:**

- Have you received any training on marking and providing feedback to students?
- Has you institution provide clear guidance on what is deemed as effective feedback? And how useful how you found this?
- How confident are you with marking student assessments and providing constructive feedback?

**3.4 Personal Tutor**

- Do you have act as a personal tutor for students?
- Did you receive any training on how to be an effective personal tutor?
- On commencing your current role did you feel you had the skillset to be an effective personal tutor?

**3.5 Curriculum Evaluation and Development:**

- Do you evaluate the effectiveness of your course curriculum and content?
- How confident do you feel at evaluating your own teaching?
- How prepared do you feel to be involved in course/curriculum development?

**Section 4:**

**4. Moving Forward:**

- Do you plan to remain in academia?
- Has the experience matched your expectations/motivations of working in Higher Education?

**Semi-Structured Interview Questions:**

1. What motivated you to step in to HE?
2. How prepared did you feel to teach students in HE when you first started? Has this changed with time?
3. Looking back at the first weeks/months in your academic role – what would have been the most useful, helpful thing to know/have had support with?
4. What has been the most challenging or unexpected part of working in academia?
5. What has been the main factor – or combination of factors, that has enabled you to settle and develop into your academic role?
6. How much do you feel a part of the academic/university community?
7. Has the experience of working in higher education matched your expectations?
8. What would your top piece of advice be to someone considering a move from clinical practice to University teaching role?
9. How would you define a gold-standard staff induction program in higher education for new starters transitioning from clinical roles?
